# Updated good practice recommendations for outpatient parenteral antimicrobial therapy (OPAT) in adults and children in the UK

**DOI:** 10.1093/jacamr/dlz026

**Published:** 2019-08-26

**Authors:** Ann L N Chapman, Sanjay Patel, Carolyne Horner, Helen Green, Achyut Guleri, Sara Hedderwick, Susan Snape, Julie Statham, Elizabeth Wilson, Mark Gilchrist, R Andrew Seaton

**Affiliations:** 1 University Hospital Monklands, NHS Lanarkshire, Airdrie, UK; 2 Southampton Children’s Hospital, University Hospital Southampton NHS Foundation Trust, Southampton, UK; 3 The British Society for Antimicrobial Chemotherapy, Birmingham, UK; 4 Blackpool Teaching Hospitals NHS Foundation Trust, Blackpool, UK; 5 Belfast Health and Social Care Trust, Belfast, UK; 6 Nottingham University Hospitals NHS Foundation Trust, Nottingham, UK; 7 South Warwickshire NHS Foundation Trust, Warwick, UK; 8 Manchester University NHS Foundation Trust, Manchester, UK; 9 Imperial College Healthcare NHS Trust, London, UK; 10 Queen Elizabeth University Hospital, NHS Greater Glasgow and Clyde, Glasgow, UK

## Abstract

UK good practice recommendations for outpatient parenteral antimicrobial therapy (OPAT) were published in 2012 and 2015 for adult and paediatric patients, respectively. Here we update the initial good practice recommendations in a combined document based on a further review of the OPAT literature and an extensive consultation process. As with the previous good practice recommendations, these updated recommendations are intended to provide pragmatic guidance for new and established OPAT services across a range of settings and to act as a set of quality indicators for service evaluation and quality improvement.

## Contents

1. Introduction
2. Methods
   2.1 Scope and purpose
   2.2 Stakeholder involvement
   2.3 Literature review
   2.4 Consensus process and guideline development
3. Recommendations
   3.1 OPAT team and service structure
      3.1.1 Formal OPAT programme
      3.1.2 OPAT ‘bundles’
      3.1.3 OPAT team composition
      3.1.4 OPAT and telemedicine
      3.1.5 OPAT in new settings
      3.1.6 Evidence gaps
   3.2 Patient selection
      3.2.1 Identification of patients
      3.2.2 Selection criteria
      3.2.3 OPAT in hard-to-reach groups
      3.2.4 Evidence gaps
   3.3. Antimicrobial management and drug delivery
      3.3.1 Continuous antimicrobial infusions
      3.3.2 Infusion devices
      3.3.3 Vascular access
      3.3.4 Antimicrobial agents
      3.3.5 Antimicrobial stewardship
      3.3.6 *Clostridioides difficile* risk in OPAT
      3.3.7 Evidence gaps
   3.4 Monitoring of the patient during OPAT
      3.4.1 General considerations
      3.4.2 Laboratory test monitoring
      3.4.3 Antimicrobial switches
      3.4.4 Evidence gaps
   3.5 Outcome monitoring and clinical governance
      3.5.1 Outcome monitoring for quality, service development and research
      3.5.2 Standard outcome measures
      3.5.3 Patient experience
      3.5.4 Evidence gaps
4. Conclusions

## 1. Introduction

Outpatient parenteral antimicrobial therapy (OPAT) has been shown to be safe and effective for a wide range of infections in adults and children,^1–7^ and is now a routine part of patient care in the UK. First described >40 years ago in the USA, it was developed in several UK teaching hospitals around 20 years ago. Since then there has been an expansion in the number of UK OPAT services, with a conservative estimate of >100 formal hospital-based services.^8^ The increase in OPAT services has been attributed to a number of factors, including financial pressures in the NHS, the focus on moving care out of acute hospitals, development of antimicrobial agents that can be administered once daily, weekly or as continuous infusions, advances in vascular access and infusion devices, and OPAT acceptance by patients and healthcare professionals.^9^^,^^10^ Furthermore, OPAT is now being actively promoted as part of the UK government’s stewardship initiatives.^11^

In the UK, OPAT is being delivered in an ever-increasing variety of clinical and non-clinical settings. In hospitals, OPAT services have traditionally been based in infectious diseases (ID) units and, less frequently, in specialist units such as those for patients with cystic fibrosis. Now, however, we are increasingly seeing OPAT services run by acute or general physicians with infection input from a clinical microbiologist, rather than the ‘traditional’ UK model of an ID physician undertaking both of these roles. New OPAT services may be established in acute medicine or emergency department (ED) ambulatory care units or based in the community.^12^^,^^13^ Notably, in a 2015 BSAC survey of UK acute medicine and ED physicians, it was reported that the majority of patients with cellulitis treated with intravenous antibiotic therapy without hospital admission were not managed within a structured OPAT service, often without daily review for intravenous-to-oral switch (R. A. Seaton, personal communication). As ambulatory care services develop, it is important that the same OPAT governance procedures are in place to ensure appropriate and safe antibiotic prescribing, particularly the involvement of a clinical microbiologist or ID consultant. In addition to increasing variation in OPAT service delivery, self-administration or carer administration is increasingly being used as a cost-efficient alternative to the infusion centre model.^1^^,^^14^^,^^15^

There has been an increase in the complexity and comorbidity of patients and in the complexity of the infections being managed, with a move away from predominantly short-course therapy towards prolonged treatment courses for bone and joint infections, endocarditis and other complex deep-seated infections.^1^^,^^4^^,^^16^^,^^17^

Over the last 5–10 years there has been increasing recognition of the important relationship between antimicrobial stewardship (AMS) and OPAT. The prudent use of antimicrobial agents is now viewed as essential to maintain the effectiveness of our antimicrobial armoury against increasing global antimicrobial resistance.^18–20^ OPAT has been recognized as playing an important role in AMS and is one of the five options for ‘focusing’ antimicrobial therapy when reviewing therapy after an initial empirical approach.^11^ Interestingly, the role of OPAT services has been extended in some areas to the supervision of complex oral antimicrobial therapies, for example weekly toxicity monitoring for patients receiving linezolid.^21^ However, OPAT also has disadvantages as regards AMS, in particular the potential use of agents with a broader antimicrobial spectrum than may be necessary due to the logistics of once daily versus multiple daily dosing regimens, or the unnecessary prolongation of intravenous therapy when oral antibiotics would be suitable.^22^ Furthermore, it is recognized that OPAT is only one element of a patient’s management and that there needs to be consideration of other aspects of care, including surgical or radiological intervention and determination of clear treatment goals, including, when appropriate, long-term suppression or palliation in incurable infection.

Since the publication of the original UK OPAT good practice recommendations (GPRs) for adults and children^23^^,^^24^ a number of other national OPAT guidelines and recommendations have been published.^25–27^ The aim of the original UK recommendations was to provide a resource for teams developing new services, as well as to provide a practical set of quality indicators for existing services. Considering the growing OPAT literature and developments in clinical practice, it felt timely to update the UK recommendations to ensure that they continue to provide appropriate guidance to OPAT services across a range of healthcare settings.

## 2. Methods

### 2.1 Scope and purpose

This update covers both adult and paediatric OPAT; specific recommendations relating to either adult or paediatric populations are highlighted. Updated GPRs are presented as a literature update following the original literature reviews, with revised recommendations for each of the five key areas used in the original GPRs. Revised or amended recommendations are depicted using italics.

### 2.2 Stakeholder involvement

The BSAC was the host organization. Working Party membership comprised consultants in adult and paediatric ID, medical microbiologists, antimicrobial pharmacists and clinical nurse specialists in paediatric ID and OPAT.

### 2.3 Literature review

The Cochrane Library issues 8 of 12 (including the Central Register of Controlled Trials), CINAHL, EMBASE, PubMed and Web of Science (Science Citation Index Expanded) databases were comprehensively searched from 1 July 2010 to 31 July 2017 (Table S1, available as Supplementary data at *JAC* Online). A further search, covering the period 1 July 2017 to 31 August 2018, was completed shortly before the consultation process to capture any additional papers published. Terms were searched and collated for adults and run again with search terms specific for paediatrics (Table S1).

**Figure 1.**
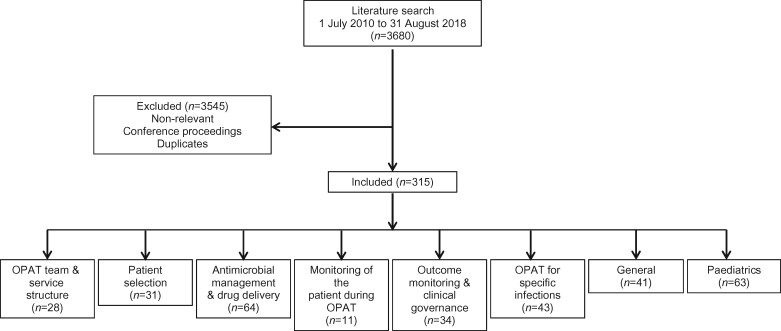
Flow diagram illustrating the process of the literature search.

A total of 3007 references were identified from the first literature search (2463 references for adults and 544 for paediatrics) and 673 references in the second search (Figure 1). An initial screen identified non-relevant references (defined as those references with no mention of intravenous antibiotics or outpatient therapy), conference proceedings and duplicate references, which were removed from further appraisal. Detailed screening using the abstract was completed by members of the core working group. Remaining references were divided into several key areas relating to the areas of the previous GPRs (Figure 1). Where references were deemed to be relevant to all key areas, they were allocated to the ‘General’ category; references related to the use of OPAT for specific infections were allocated to the ‘OPAT for specific infections’ category, and papers relating specifically to the paediatric population were categorized into a ‘Paediatrics’ category. Once references had been divided into the appropriate key groups, full-text articles in the English language were obtained and reviewed. Where evidence on a particular recommendation was lacking in the literature this was noted. As most reviewed references described non-interventional, observational studies or case series, levels of evidence have not been included in this review.

### 2.4 Consensus process and guideline development

A core working group was established (A. L. N. C., M. G., R. A. S., S. P. and C. H.). Clinicians from across the range of professional groups involved in OPAT (nursing/medical/pharmacy) were invited to provide an appraisal of the literature identified by the searches. The evidence appraisal was reviewed in detail by the core working group at a meeting in May 2018, and subsequently by telephone and e-mail communication. Changes to the initial GPRs were made and new GPR statements were written (both indicated as *italic* text). The revised recommendations were circulated to all reviewers for checking sense and to ensure that there were no omissions. Following further revision, the draft updated GPRs were sent to a comprehensive list of stakeholders and uploaded to the BSAC website (www.bsac.org.uk). A formal 4 week consultation process was completed. The GPRs were revised in the light of the comments received, with nine revisions to the recommendations.

## 3. Recommendations

### 3.1 OPAT team and service structure

#### 3.1.1 Formal OPAT programme

**Figure 2.**
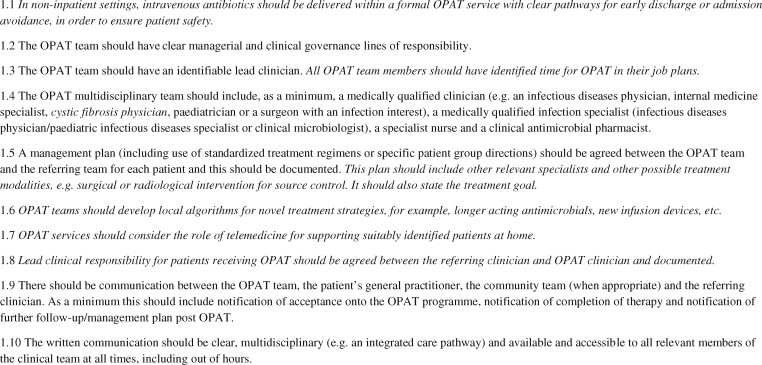
OPAT team and service structure. Text in *italics* denotes a new recommendation or a previous recommendation that has been amended.

Recommendations relating to the OPAT team and service structure are listed in Figure 2. The previous UK GPRs stressed the importance of a formal OPAT service structure with clear clinical and managerial accountability. Many recent publications supported this view.^28–32^ Heintz *et al.*^33^ prospectively estimated the effect such a strategy has on patient safety and cost-effectiveness. Of 569 referrals in 536 patients enrolled into an OPAT programme, involvement of the OPAT team resulted in safety, regimen simplification or efficacy interventions for OPAT courses in 56.1%, 40.6% and 26.8%, respectively. Interestingly, OPAT team review resulted in a significant improvement in interventions related to safety (64% versus 48%) and efficacy (36% versus 21%) but not regimen simplification, compared with those not reviewed by the OPAT team. This suggests that knowledge of antimicrobials alone will not recoup all the value of OPAT team involvement. OPAT interventions may relate to a broader holistic assessment of the patient and the infection and/or more detailed assessment of social and logistical factors.^33^ Yan *et al.*^34^ retrospectively described a Canadian cohort of 104 patients discharged to receive intravenous antimicrobials without a formal OPAT programme. Although 56 did receive post-discharge follow-up from an ID physician, 56% returned to the ED within 60 days of discharge, while 26% required readmission; 48% of the returns were due to infection relapse or treatment failure and 23% could be attributed to OPAT-related complications.^34^ The implication is that with a formal OPAT programme these return visits to the ED could be avoided with careful patient selection and appropriate and timely monitoring and intervention during follow-up.

There was further evidence that many OPAT services worldwide lack a formal service structure. Lane *et al.*^35^ conducted a survey of US ID physicians and concluded that OPAT is frequently delivered by non-specialist OPAT teams without systems for tracking adverse events or monitoring patient outcomes. A further survey of US ID physicians in 2015 reported that only 56% of respondents were part of a formal OPAT programme.^17^ Respondents reported difficulties in communication between hospital physicians and community teams delivering OPAT, and variability in blood test monitoring and follow-up.

#### 3.1.2 OPAT ‘bundles’

Several papers reported the impact of incorporating formal processes into an OPAT service. Keller *et al.*^36^ developed what they termed an ‘infectious diseases transition service’, comprising a physician, specialist nurse and pharmacist, and evaluated its impact on the care and outcomes of 488 OPAT patients. After the implementation of the transition service, readmissions decreased from 38.1% to 27.9%. However, importantly, the authors had also included a control group of patients who had been discharged on OPAT without formal ID consultation. Readmission rates also fell in this group with no significant impact of the transition service *per se*. Similarly, implementation of the transition service had no significant impact on ED visits. However, the transition team care was associated with improved process-of-care outcomes, such as fewer antimicrobial therapy errors, improved laboratory test receipt and increased follow-up visits.^36^

Similarly, Nguyen^37^ described an acute infection management service designed to transition patients with infections safely from the acute hospital setting to receive OPAT. In this study, 80 patients, of whom 66% had a diagnosis of cellulitis, received the service over 13 months, generating 618 follow-up visits. The service was safe, with only two patients requiring admission, one for fever and one for reasons of transportation.

Muldoon *et al*.^38^ and Madaline *et al.*^39^ report a more comprehensive approach to OPAT using OPAT ‘bundles’, where a bundle has been defined as ‘a set of practices that together should improve outcomes’.^40^ Muldoon *et al.*^38^ outline a theoretical approach using a bundle comprising six components, based on the IDSA guidelines and UK GPRs: (i) patient identification/selection; (ii) ID consultation; (iii) patient/family education; (iv) care transition; (v) outpatient monitoring; and (vi) OPAT programme measures.

In addition to the recommendations described in the initial UK GPRs, four further recommendations are made: (i) patients or carers should be given information about OPAT, its benefits and risks, and the potential complications and side effects of treatment; (ii) the follow-up appointment should be handed to the patient prior to discharge from hospital; (iii) a clear plan should be made for line removal at the end of therapy; and (iv) consideration should be given to developing novel approaches to patient education,^23^^,^^24^ for example mobile phone applications or simple cartoon-based educational material.^29^

Madaline *et al.*^39^ used a very similar bundle in a pre- and post- intervention study, but without a contemporaneous control group. Those patients receiving the bundle demonstrated a lower 30 day readmission rate when compared with the previous standard of care (13% versus 26.1%) and improved monitoring of blood tests and attendance at follow-up appointments, but no significant difference in ED attendances. The theoretical paper of Halilovic *et al.*^41^ on risks associated with OPAT further breaks down the bundle described by Muldoon *et al.*^38^ into key elements, designating an OPAT team member to take responsibility for each.

#### 3.1.3 OPAT team composition

The previous UK OPAT GPRs made a recommendation about the composition of the OPAT multidisciplinary team (Figure 2; Recommendation 1.4) and several more recent papers supported this recommendation. In a retrospective case-controlled study, Shah *et al.*^42^ investigated 99 OPAT patients, of whom 60 were assessed by an ID physician and 39 received non-ID physician care. Those assessed by ID physicians were 3.9 times more likely to adhere to monitoring guidance. The addition of an ID pharmacist to the non-ID physician care increased the adherence to monitoring from 35.9% to 100%, underlining the critical importance and additional value of having an antimicrobial pharmacist on the OPAT team.^42^ Shresthra *et al.*^43^ retrospectively looked at 263 potential OPAT patients referred to ID physicians over a 3 month period. In 260 of 263 episodes the authors concluded that value was added by the ID physician; antimicrobial treatment was optimized in 84%, significant patient assessment was made in 52% and an additional medical care contribution was made in 71%. In 33% of cases, an intervention was made in all three of these domains. Perhaps most critically, OPAT was deemed not necessary in 27% (60% of these patients were changed to oral antimicrobials and for 40% no antimicrobial was deemed necessary).^43^ Hersh *et al.*^44^ also demonstrated a 24% reduction in use of OPAT in a paediatric cohort following introduction of a process of expert review.

OPAT must be guided by the principles of AMS and should operate within an AMS programme.^22^ In a retrospective observational study, Hase and Hosokawa^32^ described OPAT use of ceftriaxone in a Japanese hospital without a formal OPAT team. A total of 268 patients received ceftriaxone with some courses curtailed due to readmission (10.8%) and death (4.5%). Disappointingly, ceftriaxone was used empirically in 92.2%, blood cultures were not performed in 62.3% and no cultures of any type were performed in 30%.^32^ For children managed within paediatric OPAT (pOPAT) services, there is also increasing evidence that in the absence of paediatric AMS team oversight children have higher rates of bug/drug mismatches, drug-dosing errors and readmissions, and less rigorous laboratory monitoring of drug side effects.^45^ Embedding paediatric AMS within OPAT services has been shown to reduce the duration of intravenous antibiotics, through earlier cessation of antibiotics or prompt intravenous-to-oral switching.^44^^,^^46^ This is especially relevant when children are being ambulated directly from the ED or paediatric assessment unit as part of an admission avoidance strategy.^47^

#### 3.1.4 OPAT and telemedicine

Three studies where telemedicine was used in the management of patients receiving OPAT were reviewed. Bradford *et al.*^48^ reported the use of telemedicine for remote monitoring of paediatric oncology patients receiving home intravenous therapy administered by parents in comparison with administration in the home by a visiting nurse or administration in an infusion centre. They found that the telemedicine model allowed the delivery of safe care with significant cost reduction compared with the other treatment strategies.^48^ Greenup *et al*.^49^ used telemedicine to provide support to hospital-in-the-home (HITH) nurses when discharging patients from the service. The use of telemedicine to obtain clinical advice from a hospital-based physician allowed patients to be discharged from the service without the need for in-person consultation and had no significant impact on 28 day readmissions.^49^ Thirdly, Tan *et al.*^50^ reported the use of telemedicine in the management of 88 episodes of OPAT in 83 patients over a wide geographical area around Perth, Western Australia. OPAT was initiated in hospital and ongoing treatment was administered by local nursing services, supported by a once-weekly videoconference with an ID physician. Clinical outcomes were comparable to conventional OPAT and the authors estimated that >100 000 km of travel was avoided.^50^ Telemedicine is likely to be used increasingly in future^51^ and should be incorporated into OPAT programmes systematically with appropriate plans for escalation/safety-netting.

#### 3.1.5 OPAT in new settings

OPAT in the UK has predominantly been delivered by teams based in acute hospitals. Such services tended to deliver OPAT through one or more of three models: the ‘infusion centre’ model, where patients attend an OPAT facility daily; the ‘visiting nurse’ model, where a nurse (from either primary or secondary care) delivers therapy in the patient’s home; or the ‘self-administration’ model, where the patient or a carer is taught to administer therapy with regular supervision from the OPAT service. During the early years of OPAT in the UK, OPAT services were usually run by ID units, but increasingly other specialities are setting up OPAT. In particular there are now reports in the literature of services based in acute medicine or emergency ambulatory care units. Yan *et al.*^13^ described a predominantly infection nurse-led service in a British hospital where access to medical care was via the nurse through the ED physicians. In this retrospective cohort study, 140 patients received OPAT, either returning to hospital daily (*n* = 94) or in their own home through a district nursing visit (*n* = 46). The service was safe, with a failure/complication rate of 5.7% and hospital readmission rate of 3.6%. The mean duration of OPAT was 4.4 days, with the predominant diagnosis being cellulitis.^13^

In addition to increasing diversity in OPAT providers in secondary care, another key development has been the establishment of OPAT based within primary care organizations and delivered in the community. Antimicrobial therapy may be initiated and carried out exclusively in the community, or alternatively may be initiated in hospital and transferred to a community-based OPAT service.^10^ Several papers describe such services.^12^^,^^52–55^ Nazarko^12^ outlines the advantages and potential disadvantages of developing a specialist intravenous therapy team or incorporating OPAT into the day-to-day activity of established community nurses. A dedicated intravenous therapy team would provide enhanced expertise in management of different devices and antimicrobial agents and in practical skills such as venepuncture and cannulation. Furthermore, it may be easier to train a small team to recognize clinical deterioration. Gray *et al*.^56^ used patient scenarios to investigate how HITH nurses recognize and respond to the deteriorating patient; however, service capacity may be limited by a small team, particularly over large geographical areas. The use of the larger pool of community nurses would provide greater capacity and also allow nurses to deliver more than one type of care to housebound patients at the same visit, for example wound or ulcer dressings and insulin injections. However, community nurses may be unfamiliar with intravenous therapies and require theoretical and practical training; there is also the issue of maintaining competency. Whatever the model for antimicrobial administration, as with services based in secondary care, the involvement and oversight of a full OPAT team is essential, including a lead OPAT nurse, antimicrobial pharmacist and infection specialist.^55^^,^^57^ It is also essential that a responsible physician is clearly identified for every patient; this may be a primary care doctor or hospital specialist. Mace *et al.*^58^ examined the impact of introducing a dedicated OPAT team to a paediatric HITH service in Australia and reported improved adherence to monitoring guidelines, reduced readmissions and fewer patients on prolonged antimicrobial therapy, demonstrating the importance of medical governance in a nurse-led service.

In establishing a community-based OPAT service, key issues to consider include workload and capacity, dose frequency, use of boluses or infusions, line insertion and care, and arrangements for prescribing and dispensing antimicrobials, for clinical reviews and for escalation if complications occur. Several authors reported the use of ‘OPAT kits’ made up by local pharmacy manufacturing units and containing all medications, diluents and consumables required for a week, tailored to each patient and type of vascular access device.^10^^,^^12^^,^^52^^,^^57^ Barker and Lyden-Rodgers^55^ described an audit of 26 patients receiving intravenous antibiotics in their homes and concluded that cost savings in nursing time may be possible if single as opposed to paired visiting community nurses deliver bolus doses in comparison with infusions. Although community intravenous therapy services may deliver a range of parenteral agents, such as bisphosphonates, iron, chemotherapy or blood products, involvement of an infection specialist and antimicrobial pharmacist in the care of patients receiving intravenous antibiotics is essential in ensuring that AMS is prioritized. Outcome monitoring in general may be more challenging in the community context where patients are under different primary care physicians and teams of nurses, and also needs to be considered when establishing a community-based service.

#### 3.1.6 Evidence gaps

One key evidence gap relates to the appropriate time commitment for OPAT team members. Financial pressures within healthcare systems may have an impact on the number of staff being employed to deliver OPAT; data on the appropriate ratio of patients to OPAT specialist nurses in particular would be helpful to justify the funding required to deliver an OPAT service. Review of the UK BSAC OPAT National Outcomes Registry System (NORS) (http://opatregistry.com) in 2018 suggested that for established hospital-based infection-led OPAT services, the average establishment per 100 episodes per year was 1.5 whole-time equivalents (WTE) for nursing, 0.3 WTE for medical staff, and 0.25 WTE for specialist pharmacists (M. Gilchrist, personal communication). There are currently no data available for community-based OPAT services.

### 3.2 Patient selection

#### 3.2.1 Identification of patients

**Figure 3.**
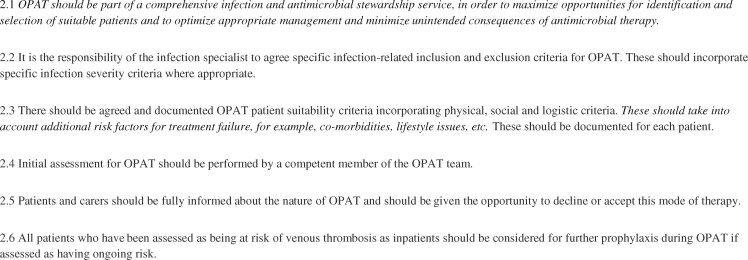
Patient selection. Text in *italics* denotes a new recommendation or a previous recommendation that has been amended.

Recommendations relating to patient selection for OPAT are listed in Figure 3. In the past, identification of suitable patients for OPAT was often through direct referral from inpatient teams or *ad hoc* referrals from other infection specialists. With increasing experience of OPAT and demonstration of its safety and patient focus, there is a realization that we must move from a passive or ‘opportunistic’ approach to identifying suitable patients to a more active approach. Examples include participation in multidisciplinary clinical meetings in key specialties such as the diabetic foot service or orthopaedics. O’Hanlon *et al.*^52^ describe 14 patients with diabetic foot infections, suggesting that nurses with OPAT training can be useful case finders for patients who may be suitable for OPAT within the diabetic clinic.

Patients may also be identified as potentially suitable for OPAT actively through infection initiatives such as bacteraemia reviews and antimicrobial ward rounds. One study demonstrated an increase in the numbers of patients receiving OPAT through the use of a bacteraemia database to identify patients who may be suitable for OPAT once stable.^59^ Dryden *et al.*^60^ describe infection team review of inpatients receiving antimicrobial therapy in six UK hospitals. Of 89 patients who were suitable for discharge, 55 were suitable for oral outpatient treatment, 24 had their antibiotics stopped and 10 would have required OPAT. This study demonstrated the value of an infection team in identifying patients appropriate for OPAT, but more significantly in recommending the use of oral agents over intravenous therapy where clinically appropriate. Conant *et al*.^61^ also reiterated the importance of involvement of an infection specialist in optimizing antimicrobial therapy, ensuring appropriateness of patients for OPAT and contributing to AMS.

#### 3.2.2 Selection criteria

As with the previous GPRs, many papers concluded that careful patient selection was critical to improving outcomes and reducing risks surrounding OPAT. Selection involves consideration of patient-specific criteria, such as ability to understand and consent to OPAT, likelihood of compliance, appropriate home circumstances, ability to attend for OPAT, support from family members and safety of visiting healthcare staff. Having no primary care provider has also been found to be a risk factor for OPAT complications.^62^^,^^63^

Infection-related criteria are also important in patient selection, for example the site and severity of infection, presence of complications of infection, prior duration of antimicrobial therapy, initial response to treatment and availability of oral antibiotic options.^64^^,^^65^ Underwood *et al.*^66^ reported that of 781 patients referred to their OPAT service, 31% were assessed as not requiring intravenous therapy following review of their proposed management plan.

There is also increasing evidence that patient selection should move beyond application of rigid criteria but should take into consideration additional factors that may influence the likelihood of OPAT failure or complications. Schmidt *et al.*^67^ found that the risk of unplanned hospitalization in OPAT patients was increased in older patients with more comorbidities: for each additional point in the Charlson comorbidity index the risk of unplanned readmission increased by 5%. They also found that the risk of unplanned hospitalization varied depending on the type of facility in which patients were receiving OPAT. It is not clear whether this relates to patient factors determining the need for a specific OPAT facility or to factors relating to the facility itself, for example expertise of staff or robustness of arrangements for monitoring blood tests.^68^ Whatever the reason, Schmidt *et al.*^67^ suggested that the Charlson comorbidity index may be useful both in patient selection and in determining the most appropriate site or model of OPAT. Duncan *et al.*^69^ retrospectively reviewed 80 episodes of OPAT for infective endocarditis and found that on multivariate analysis cardiac or renal failure were independently associated with OPAT failure. Similarly, Seaton *et al.*^70^ found that the presence of diabetes or vascular disease was a predictor of poorer outcomes in 963 patients with skin and soft tissue infection managed via OPAT. However, Allison *et al.*^71^ found an association between 30 day readmissions and four factors—increasing age, use of aminoglycosides, presence of antibiotic-resistant organisms and the number of prior hospitalizations in the preceding year—but found no impact of comorbidities. These findings may have been due to differences in population sizes or in the screening protocol for acceptance for OPAT and demonstrate the difficulty in comparing studies and also the need for further work to look at predictors of poor outcomes in OPAT.

In assessing the appropriateness of a patient for OPAT there may also be considerations relating to their longitudinal progress. As an example, a recent review of the paediatric cystic fibrosis literature confirmed that no randomized controlled trials have been conducted comparing inpatient versus OPAT management of children with cystic fibrosis.^2^ Cohort studies have yielded conflicting results in terms of patient/parent satisfaction and clinical outcomes.^72–74^ It is difficult therefore to offer clear recommendations about the safety and effectiveness of pOPAT in children with cystic fibrosis, and the authors suggest a more holistic view of the likely benefits to the child/young person/family regarding the potential impact of pOPAT on their long-term respiratory function.

#### 3.2.3 OPAT in hard-to-reach groups

OPAT was offered to patients with mental health diagnoses, people who inject drugs (PWIDs) and homeless patients by a few centres. Ho *et al.*^75^ describe successful treatment of 29 PWIDs at their centre in Singapore; however, they used strict selection criteria and standardized measures to prevent and detect line misuse, including tamper-detectable line dressings. Beieler *et al.*^76^ used OPAT successfully for treatment of infections in homeless people, most of whom were PWIDs; however, OPAT was delivered in a medical respite facility with close supervision, an overnight curfew and access to substance misuse services and opioid replacement therapy. The importance of careful patient selection was highlighted by several authors;^77–79^ Camsari and Libertin^77^ concluded that they would offer OPAT to PWIDs only if they had been abstinent for >12 months. Buehrle *et al.*^80^ stressed the importance of a comprehensive support package; in their study only 39% of PWIDs completed OPAT successfully due to a combination of clinical and social factors. They, and others, have concluded that recent or ongoing injection drug use may be considered a contraindication to OPAT and that there was a need for further research into the reasons for the high rates of OPAT failure, the effectiveness of oral therapy as an alternative to OPAT, and the benefit of residential addiction treatment alongside OPAT.^80^^,^^81^ Hernandez *et al*.^82^ also identified social factors, such as missed appointments or loss of temporary accommodation, as important in success or failure of OPAT in their retrospective study of 43 homeless people, 33 (77%) of whom completed treatment successfully. Longer-acting antimicrobial agents may also be of benefit in PWIDs and other hard-to-reach groups to reduce the reliance on patient compliance with daily therapy and to avoid long line placement.^83–86^

#### 3.2.4 Evidence gaps

As noted above, more prospective research is required to enable us to predict more accurately which patients are most likely to have a successful, or unsuccessful, outcome of their OPAT episode. There is also a paucity of paediatric data on patient selection and risk stratification. Although there is always a need for clinical judgement, it would be helpful to develop evidence-based algorithms to support patient selection in future.

The previous GPRs included a recommendation on risk assessment for venous thromboembolism (VTE) in patients undergoing OPAT following an inpatient stay. The updated literature review identified one paper on the risk of VTE with OPAT.^87^ This was a retrospective review of 780 OPAT episodes over a 3 year period; no patients received VTE prophylaxis. Two patients developed deep vein thrombosis within 90 days of OPAT, giving a VTE incidence of 0.26%. The authors concluded that patients commencing OPAT should not be assessed routinely for VTE prophylaxis using an inpatient algorithm. Although these data are reassuring, patients on OPAT do have significant infections and are therefore at increased risk of VTE compared with the general population^88^ and it would be useful to have further prospective data on the risk of VTE and the optimal assessment strategy for prophylaxis.

### 3.3 Antimicrobial management and drug delivery

#### 3.3.1 Continuous antimicrobial infusions

**Figure 4.**
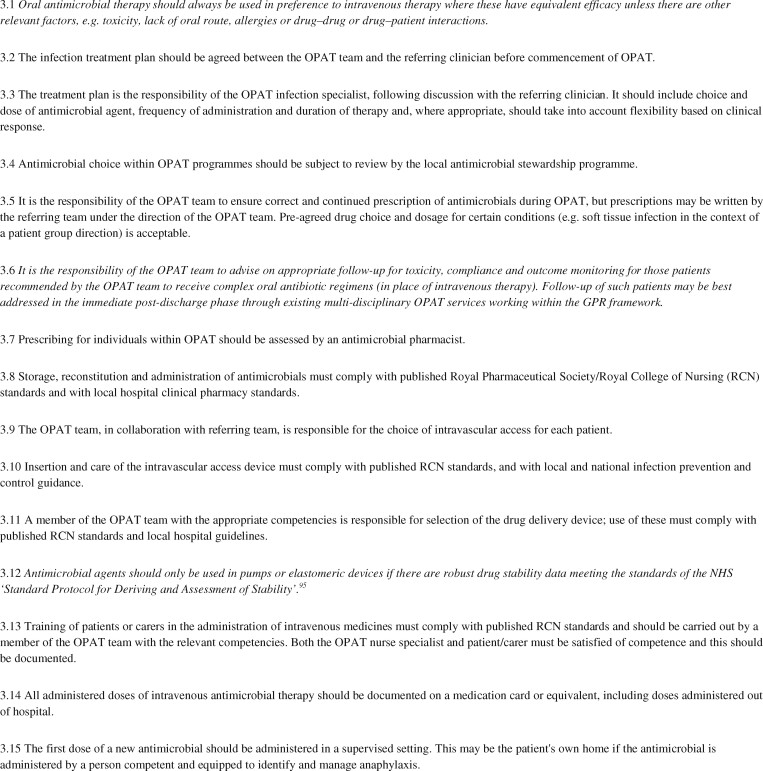
Antimicrobial management and drug delivery. Text in *italics* denotes a new recommendation or a previous recommendation that has been amended.

Recommendations relating to antimicrobial management and drug delivery in OPAT are listed in Figure 4. Four papers presented data on the use of continuous infusions of amoxicillin, meropenem, vancomycin and clindamycin, respectively, presenting data on clinical outcomes and drug stability.^89–92^ All reported relative success; however, there were questions over antibiotic degradation and the potential need for therapeutic drug monitoring. Voumard *et al.*^93^ also reported that OPAT using elastomeric pumps for the continuous administration of four antibiotics (flucloxacillin, cefepime, vancomycin and piperacillin/tazobactam) was efficacious and safe. Drug concentration measurements, considered a proxy for efficacy, confirmed adequate circulating antibiotic exposures consistent with the observed high rate of therapeutic success.

Within the wider OPAT arena concerns have been expressed about the lack of antimicrobial stability data, particularly within elastomeric devices.^22^ The UK BSAC OPAT initiative conducted a separate literature review into antimicrobial stability within elastomeric devices.^94^ It found no published studies that comply with UK national standards for stability testing.^95^ As a result of this work an antimicrobial stability testing work stream was created within the UK BSAC OPAT initiative. Two stability studies have been published to date. Flucloxacillin was demonstrated to be chemically stable when reconstituted with 5% citrate buffer for up to 14 days of storage and for an additional 24 h at ‘body worn’ temperature. Flucloxacillin is therefore suitable for extended infusion via an elastomeric device within an OPAT setting.^96^ In contrast, meropenem showed significant degradation with or without buffering and so is not suitable for continuous infusions in the OPAT setting.^97^ BSAC drug stability testing studies are open-access and designed to allow OPAT services to use these agents where the clinical need exists (http://www.bsac.org.uk/drug-stability-testing-programme/).

#### 3.3.2 Infusion devices

More generally, some papers explored the use of new devices. Oliver^98^ reviewed benefits and disadvantages of elastomeric devices and described experience with one type of elastomeric device, with very positive nurse evaluations. Saillen *et al.*^14^ reported high levels of patient satisfaction with elastomeric devices, particularly from patients who were self-administering via an elastomeric device, as opposed to those who had devices changed by visiting nurses or at the OPAT unit. Hobbs *et al.*^99^ present the protocol for a study to evaluate patient and nurse satisfaction with electronic and elastomeric portable infusion pumps used at home (CHID study), which may be useful in guiding their further use.

In contrast, Pandya *et al.*^100^ reported 14 adverse events in 10 of 31 patients receiving intravenous antibiotics by elastomeric device infusion. Five adverse events were related to the infusion device, including device failure and leakage. These adverse events resulted in additional telephone calls and nurse visits. This reinforces the need for a robust OPAT service structure with escalation pathways, including flexibility to provide specialist patient input when needed, rather than defaulting to the ED, where staff may be unfamiliar with the device.^101^

#### 3.3.3 Vascular access

The literature search identified only one paper relating to vascular access in adults. Bedford and Waterhouse^102^ described the development of an out-of-hospital nurse-led service for insertion of peripherally inserted central cannulae (PICCs) using ECG-guided central line tip placement. This new technology removes the need for radiological or fluoroscopic confirmation of the correct position of the PICC tip within the lower third of the superior vena cava, and therefore the need for patients to attend hospital for their procedure. The authors described the process for doing this, including patient consent and safety measures, and reported 55 successful community-based insertions without complications. The ability to insert long-term venous access in a community setting will facilitate the expansion of community-based OPAT, as described in the service structure section above.

Four papers reviewed line complications in adults.^66^^,^^103–105^ Barr *et al.*^103^ retrospectively analysed line infections and other line events in 854 patients with midline catheters, PICCs or tunnelled central venous catheters. Incidence of line-related complication was 3.6 per 1000 intravenous catheter days, the majority of which were not infection related. Incidence of line infection was 2.3% (0.53 per 1000 intravenous catheter days) and on multivariate analysis was associated with duration of intravenous line placement with a 1.2% odds increase per additional day of intravenous therapy. Line type (midline versus central line) was not independently associated with risk of infection. Incidence of other line events (including phlebitis, leakage, extravasation, occlusion or unplanned removal) was 14.6%. As with previous studies, patient self-administration of OPAT was not associated with an increased risk of line complications, although use of multiple daily dosing of flucloxacillin and use of a midline versus a tunnelled central line were.^103^ Shrestha *et al.*^105^ reported line complications in 9% of OPAT courses, most frequently line occlusion, with increasing risk of complications with duration of OPAT. Line infection occurred in <1% of OPAT courses overall. Lam *et al.*^104^ reported that prolonged OPAT, use of double lumens and administration of benzylpenicillin and cloxacillin appeared to increase the risk of PICC occlusion, and suggested that these factors be considered by OPAT teams when choosing lines and therapeutic agents. Finally, Underwood *et al.*^66^ described 544 OPAT episodes, 5.9% of which were complicated by line-related complications (5.7 per 1000 intravenous catheter days). Most complications were non-infectious. In contrast to other published studies, the authors noted that self-administered antimicrobials were more likely to be associated with vascular device-related complications. As in other studies, non-radiologically inserted midline catheters were associated with higher rates of complications.

In pOPAT the data suggest that PICC line complications are less common than previously reported.^106^^,^^107^ Recent studies have described an 8%–15% complication rate for PICC lines used for the administration of intravenous antibiotics to pOPAT patients; infections are responsible for <25% of these adverse events.^7^^,^^108^^,^^109^ More data are required about the rate of adverse events associated with midline catheters before they can be routinely recommended for use within pOPAT services. A complication rate of 43% has been described in one small study.^110^ A dedicated paediatric intravenous line service may help to provide safer and more patient-centred intravenous access.^111^ Long-term vascular access, such as totally implantable venous access devices (TIVADs), may be indicated in some patient groups.^112^^,^^113^

#### 3.3.4 Antimicrobial agents

Several papers identified new and existing antimicrobial agents being used within the OPAT setting. One of the key areas of growth that represents a step change in antimicrobial therapy via OPAT has been the development of long-acting semi-synthetic glycopeptides, such as oritavancin^83^^,^^86^^,^^114^ and dalbavancin.^85^^,^^115–117^ These agents may be particularly useful for patients who may not otherwise be suitable for OPAT, for example where there are concerns about compliance issues or line misuse. They could also be useful in OPAT services with limited capacity due to their infrequent administration and impact on nurse workload. In using these longer-acting agents the challenge for OPAT services will be to develop clear clinical pathways and individual management plans, ensuring adequate oversight of clinical progress.^84^ Televancin,^118^ tedezolid^119^ and echinocandins^120^ are other new agents that may be used increasingly in the future.

There has been a growth in the literature surrounding more traditional antimicrobial therapies within OPAT, mainly around dosing and adverse events. Experience has grown with daptomycin,^121–124^ teicoplanin^125^^,^^126^ and ertapenem.^127–129^ Adverse events have been reported with ertapenem and tigecycline,^128–130^ adding to the literature around ensuring there is close monitoring and follow-up of OPAT patients.

#### 3.3.5 Antimicrobial stewardship

The OPAT team is integral to the development of the antimicrobial plan and judgement is required regarding the use of broader-spectrum once-daily agents to facilitate OPAT where a narrower-spectrum agent with multiple daily doses would be used in an inpatient setting.^22^ The use of continuous infusion pumps and elastomeric devices may provide a solution to this, as long as robust stability data exist. Where multiple daily doses are being given it is important that the pharmacodynamics of the antimicrobial agent are not compromised by suboptimal timing of doses through logistical constraints of the OPAT service. New long-acting agents may be advantageous in terms of logistics of administration but must be integrated into the OPAT service in a systematic way, as described earlier.

Optimal AMS also includes timely switch from intravenous to oral antibiotics (Table 1) and this should be considered both at the point of referral to OPAT as well as during a course of OPAT. General principles that should be considered when deciding whether the switch from intravenous to oral antibiotics is suitable include assessing the clinical condition, ability to absorb oral antibiotics, and availability of an appropriate oral choice. For children, dosing frequency and taste of oral suspensions should also be considered. McMullan *et al.*^139^ reviewed current evidence on duration of intravenous antimicrobials and optimal timing of intravenous-to-oral switch in children. Availability of OPAT may paradoxically result in excessively long intravenous antimicrobial courses unless subject to AMS;^2^^,^^58^^,^^140^ comparisons of antibiotic durations for specific pathologies between OPAT centres may provide useful information to guide clinical practice where evidence about the timing of intravenous-to-oral switch is lacking.^141^ The antimicrobial pharmacist plays an important role in assessing the pharmacokinetic/pharmacodynamic applicability of oral agents, potential drug–drug and drug–host interactions, antibiotic compliance, potential adverse events and monitoring needs, and how these are best addressed in an out-of-hospital setting. Patients should be counselled when antimicrobials are initiated, switched to another intravenous agent and stepped down to oral therapy.

**Table 1.** Evidence for oral versus intravenous antimicrobial therapy in selected infections

Infection type (population)	Evidence
Bone and joint infections (adults)^131^	Multicentre UK-wide randomized study of oral versus intravenous antibiotic treatment for bone and joint infections (OVIVA). In a heterogeneous group of patients with device-related and non-device-related bone and joint infection who had received <7 days of initial intravenous therapy, randomization to carefully selected oral antibiotic therapy was found to be non-inferior to continuation of intravenous therapy, with 86% success observed in both groups at 1 year. In addition, significantly lower rates of line-related complications and lower treatment costs were observed in the oral treatment group.
Bone and joint infections (children)^132^^,^^133^	Increasing evidence that pOPAT is only indicated for a minority of children with bone and joint infections. The majority of patients should be managed with an early intravenous-to-oral switch.
Endocarditis^134^	Clinically improved patients with endocarditis were randomized to early intravenous-to-oral switch or standard therapy with exclusively intravenous antibiotics. Early transition to oral therapy was found to be non-inferior to intravenous therapy. This study population would be typical of the group usually managed via OPAT; therefore, appropriate oral therapy may be a suitable alternative to OPAT for selected low-risk patients.
Intra-abdominal infection^135^	Oral antibiotics had equivalent outcomes and incurred lower costs than intravenous antibiotics following appendicectomy.
Lower urinary tract infections (adults)^136^	Non-inferiority of oral fosfomycin compared with intravenous ertapenem for the treatment of lower urinary tract infections caused by ESBL-producing Enterobacteriaceae.
Pyelonephritis (children)^137^	No difference between oral antibiotics (10–14 days) and intravenous antibiotics (3 days) followed by oral antibiotics (10 days) with respect to duration of fever or subsequent renal damage.
Pleural empyema (children)^138^	Discharge on intravenous antibiotics offers no benefit over discharging children with empyema on oral antibiotics.

#### 3.3.6 Clostridioides difficile risk in OPAT

The use of broad-spectrum agents such as ceftriaxone or ertapenem raises concern about the risk of *Clostridioides difficile*-associated diarrhoea (CDAD). The literature review identified several studies that examined the risk of CDAD with OPAT in adults. Aberdein and Chapman^142^ cross-referenced OPAT and hospital microbiology databases to identify patients who had had both OPAT and CDAD over a 5 year period. Among 1514 patient episodes and 16 750 OPAT days, 13 patients developed CDAD between 2 days of commencing and 84 days of ceasing OPAT. All but one patient had risk factors other than OPAT for CDAD, including prior hospitalization and oral antibiotics from their GP. The rate of ‘definitely OPAT-attributable CDAD’ was equivalent to six cases per 100 000 OPAT days. The comparable rate for hospital inpatients nationally at that time was 54 per 100 000 bed days.^142^ Duncan *et al.*^143^ reviewed use of ceftriaxone for OPAT and concluded that CDAD occurred in ∼0.1% of OPAT episodes. A study in the USA reported five cases of CDAD in a cohort of 681 patients, an incidence of <1 per 1000 patient-days.^144^ All five patients had had prior hospitalization and four were on concomitant acid-suppressive therapy.

#### 3.3.7 Evidence gaps

As noted above, there is a need for further data on antimicrobial stability over prolonged periods in elastomeric devices or infusion pumps, and the Drug Stability Testing work stream of the BSAC OPAT UK Project will add to existing knowledge in this area. The updated literature search provided no new data on the safety of administering the first dose of antibiotic in the home setting. However, with the growth of OPAT services based entirely in the community there is increasing evidence of the safety of this approach, as long as the nurse administering therapy is trained and equipped to manage adverse reactions, including anaphylaxis.

### 3.4 Monitoring of the patient during OPAT

#### 3.4.1 General considerations

**Figure 5.**
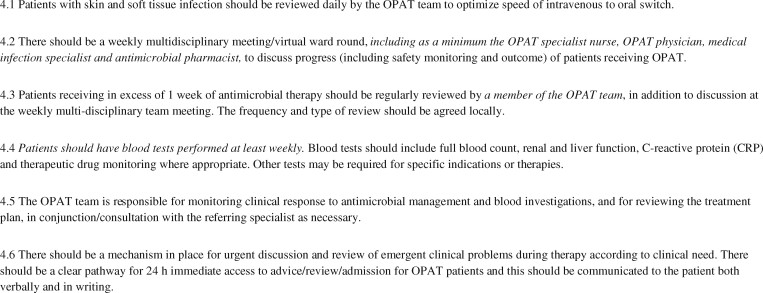
Monitoring of the patient during OPAT. Text in *italics* denotes a new recommendation or a previous recommendation that has been amended.

Recommendations relating to monitoring patients during OPAT are listed in Figure 5. There were only a small number of new papers (*n* = 11) relating specifically to monitoring patients during OPAT, although papers from other sections also provided useful new data for this domain. The risks associated with OPAT are well described:^23^^,^^24^ overall at least 25% of patients on OPAT will develop complications of therapy, ranging from mild adverse reactions to life-threatening line infections. A high proportion of patients experience risks associated specifically with prescription of their antimicrobial agents, including potential drug interactions, issues with therapeutic drug monitoring and the need for dose changes associated with changes in renal function, and the importance of a multidisciplinary approach to monitoring, with the inclusion of a pharmacist, has been emphasized.^42^^,^^145^

The previous GPRs stated that patients with skin and soft tissue infections should be reviewed daily to ensure that they are switched from intravenous to oral antibiotics as soon as this is clinically appropriate. This remains an important recommendation. Kameshwar *et al.*^140^ undertook a health-economic study of patients managed via HITH in Australia. They found that patients managed through HITH had a longer median duration of intravenous therapy than equivalent inpatients (7.5 versus 5.8 days, respectively) and that this difference offset any financial savings associated with home therapy. They speculated that the longer duration of antimicrobial therapy in HITH could have arisen due to less frequent clinical reviews and also the possibility that clinicians in hospitals were under greater pressure to free hospital beds while HITH clinicians may have adopted a more risk-averse approach.^140^

Paediatric data show that significant adverse events due to antimicrobials are infrequent in pOPAT. Readmissions due to drug side effects occurred in only 0%–2.3% of patients described in two recent pOPAT cohorts.^7^^,^^110^ However, a retrospective case series of children managed between 2008 and 2015 describes a 13.5% readmission rate due to antimicrobial side effects.^108^ Oxacillin was associated with significantly higher rates of adverse drug events (transaminitis, fever and rash) compared with ceftriaxone. High rates of adverse drug events have also been described with piperacillin/tazobactam (fever, transaminitis, neutropenia and rising inflammatory markers), with 26% of children readmitted due to drug side effects in that cohort. Adverse events occurred after a minimum of 14 days of treatment in 93% of cases.^146^

#### 3.4.2 Laboratory test monitoring

Lack of availability of recommended laboratory tests has been shown to be an independent risk factor for increased readmission rates for OPAT patients.^68^ Keller *et al.*^147^ prospectively analysed adverse drug events in a cohort of 339 patients discharged to OPAT from two academic centres; 18% developed an adverse drug event, and such events were more likely to occur within the first 14 days of treatment. However, other groups reported increasing risk of adverse events with increasing duration of intravenous antimicrobial therapy. Briggs *et al.*^148^ reported late-onset reactions to β-lactam antibiotics: 11 out of 163 patients developed symptoms such as fever, rash or abdominal pain during drug administration, or laboratory abnormalities including thrombocytopenia, leucopenia or abnormal liver function tests, with a median duration of therapy of 25 days prior to development of the adverse event. Severe neutropenia is a late complication of ceftriaxone, usually occurring after 28 days of therapy.^149^ Weekly monitoring of full blood count may also detect developing eosinophilia, which is a predictor of hypersensitivity reactions: Blumenthal *et al.*^150^ identified eosinophilia in 210 of 824 (25.5%) patients receiving OPAT, with a median time to eosinophilia of 15 days (IQR 8–22 days).

The previous GPRs recommended weekly blood-test monitoring for short-term OPAT patients but did allow a reduction in frequency of monitoring to twice monthly for longer-term stable patients. However, given evidence of increasing adverse events with treatment duration and the importance of early detection of these adverse events, the new recommendation is that all OPAT patients have blood test monitoring at least weekly regardless of treatment duration.

#### 3.4.3 Antimicrobial switches

Lee *et al.*^151^ reviewed outcomes of OPAT with β-lactam antibiotics. In 400 OPAT courses, antibiotic switches were required in 50 episodes, of which 37 were accomplished without readmission. The authors stressed the importance of close monitoring and the involvement of the infection specialist in optimizing antimicrobial therapy to minimize patient morbidity and the need for readmission.^151^

#### 3.4.4 Evidence gaps

Further information is required regarding the efficacy and potential adverse reaction profile of prolonged use of the newer long-acting semi-synthetic glycopeptides and optimal monitoring strategy. This is also true for other new agents. Another interesting area relates to patient involvement in monitoring, both in terms of awareness and early reporting of symptoms and also patient access to results through new web-based systems such as those used in diabetes or renal medicine. One key aim of OPAT is personalized, person-centred care, and involvement in monitoring their therapy may be useful in promoting patient engagement in their care and also contributing to earlier detection of adverse reactions.

### 3.5 Outcome monitoring and clinical governance

#### 3.5.1 Outcome monitoring for quality, service development and research

**Figure 6.**
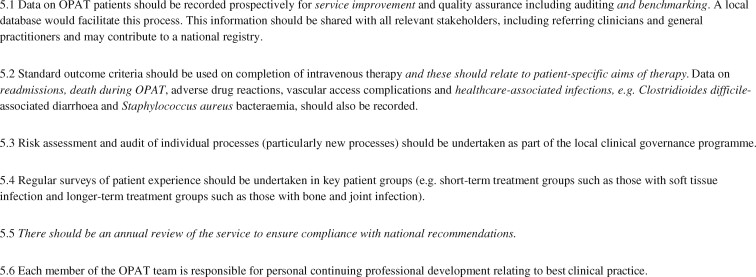
Outcome monitoring and clinical governance. Text in *italics* denotes a new recommendation or a previous recommendation that has been amended.

Recommendations relating to outcome monitoring and clinical governance associated with OPAT are listed in Figure 6. Regular reviews of the OPAT service are essential to review the activity of the service and to benchmark it against national guidelines and GPRs, and other OPAT centres and registries. This is only possible if data are collected prospectively at the level of the individual patient, using an electronic database or online outcome registry. In addition to clinical outcomes such as response to treatment and adverse events, it is also important to collect data on the OPAT episode, for example patient demographics, antimicrobial agent(s) used, duration of treatment, method of OPAT used, type of line and infusion device. It may also be useful to record other data for the purpose of local service development, for example the number of episodes and OPAT days, type of transport used by OPAT patients, time taken for antimicrobial administration and other interventions performed during OPAT attendance (e.g. podiatry review, dressing changes on ulcers and monitoring of anticoagulation).

The importance of effective AMS is now well recognized and a key UK government and NHS priority.^11^^,^^60^ Gilchrist and Seaton^22^ reviewed AMS as it applies to OPAT and proposed an OPAT AMS checklist, comprising checks relating to the individual patient, OPAT antimicrobial use, staffing and links to organizational governance procedures and policies. Again, there is a need for prospective data collection to quality-assure OPAT services from an AMS perspective.

Finally, collection of rich prospective patient data provides the opportunity to study factors influencing patient outcomes such as those described earlier (section 3.2, Patient selection). It is clear that we do not fully understand predictors of success or failure of OPAT and this must be a priority for further prospective research.

#### 3.5.2 Standard outcome measures

The previous OPAT GPRs suggested that it would be useful to develop standardized outcome measures for OPAT. These outcome criteria were divided into patient infection and OPAT service-related outcomes and have been used by UK OPAT centres and the BSAC OPAT NORS to drive improvement and allow benchmarking exercises. However, there remains a lack of standardization and clarity as to which outcomes are measured. For example, a patient may be switched from one antimicrobial to another due to a recognized side effect. This is managed as part of the OPAT management plan, resulting in successful treatment. Under the previous outcome recommendations this would be classed as partial success. The authors also recognized that patient outcomes are very much dependent on the individual treatment aim. This is particularly relevant where short- or longer-term control of the infection is the only realistic outcome of therapy. The NORS outcome measures include ‘death’ as both a patient infection ‘failure’ and an OPAT ‘failure’, which may not be appropriate where the aim of OPAT for that individual is palliation or long-term suppression. The authors therefore concluded it would be helpful to include outcomes for those clinical episodes where cure is not achievable.

Here we propose new treatment aims to be considered at the outset of an OPAT treatment course and updated OPAT outcomes (Table 2). In addition, there is increasing literature to suggest that OPAT adverse events should be reviewed in line with local organizational AMS programmes, for example healthcare-associated infections such as CDAD and bloodstream infections. In addition to patient outcomes, as in the last GPRs, it is also recommended that OPAT teams monitor specific adverse outcomes (see Recommendation 5.2).

**Table 2.** Proposed treatment aims and OPAT service outcomes

	Description
Treatment aim	
cure	To complete an agreed OPAT duration of therapy on either intravenous and/or complicated oral antimicrobials^a^ with **no** requirement for long-term antimicrobial therapy.
improvement	To complete an agreed OPAT duration of therapy on either intravenous and/or complicated oral antimicrobials (a) as part of an agreed surgical infection management plan with further surgery planned or (b) where there is a requirement for subsequent long-term or an extended course of oral suppressive antimicrobial therapy, or (c) where potentially infective prosthetic material is still *in situ*.
palliation	To undertake a course of OPAT on either intravenous and/or complicated oral antimicrobials where there are agreed ceilings of care due to comorbidities, with death being the likely outcome.
OPAT outcome	
treatment aim attained—uncomplicated	Completed OPAT therapy as per treatment aim with: **no** unplanned changes in antimicrobial agent. **no** adverse events. **no** planned or unplanned readmission related to the current OPAT episode. **no** readmission of ≥24 h for unrelated event (i.e. day case/overnight stay for another medical problem allowed).
treatment aim attained—complicated	Completed OPAT therapy as per treatment aim but **with** one or more of the following: unplanned changes in antimicrobial agent. any adverse event including readmission for <24 h related to the current OPAT episode.
treatment aim not attained	failure to complete planned OPAT therapy for any reason other than readmission due to unrelated event. worsening of infection requiring readmission. readmission for ≥24 h for any cause related to OPAT, including adverse events.
indeterminate	Readmission for ≥24 h due to unrelated event.
death	Death due to any cause, except palliation.

a Complicated oral antimicrobials refers to oral regimens that require specific monitoring or are associated with particular risk of toxicity.

#### 3.5.3 Patient experience

A major advantage of OPAT has always been the opportunity to tailor treatment regimens to individual patients, taking into consideration their circumstances and preferences. Although many studies report patient experience surveys, there has been a lack of in-depth qualitative analysis of the experiences and views of OPAT patients and family members, until two recent publications.^152^^,^^153^ Castor *et al.*^152^ undertook 37 qualitative interviews with members of 12 families of children receiving home therapies. They identified three essential themes—(i) strengthening family life; (ii) promoting health; and (iii) creating alliances—and stressed the importance of developing good relationships between family, home care service and hospital, and of paying close attention to the needs of each family member to ensure a positive experience for all.^152^ Twiddy *et al.*^153^ also undertook semi-structured interviews with 28 adult OPAT patients, as well as a focus group of 4 patients. They identified two key themes on qualitative analysis. The first comprised functional aspects of care, including the subthemes of ‘being at home but not well’, ‘convenience and flexibility’, ‘location of care’ and ‘is it safe?’ The second theme was relational aspects of care; one important element of this was a desire amongst patients for clear communication with staff who knew them to give patients confidence to collaborate in their own care. The authors then used a discrete choice experiment to identify overall patient preferences regarding OPAT. Although the most favoured model of OPAT was the model of the visiting nurse administering therapy at home, there was sufficient heterogeneity for the authors to conclude that services should ideally offer a range of OPAT delivery models.^153^

#### 3.5.4 Evidence gaps

Although there are some recent publications focusing on patients’ experiences and perspectives of OPAT, there remains a need for further research in this area, particularly relating to patient self-administration using elastomeric devices and portable infusion pumps. Further quality-of-life studies comparing OPAT with hospitalization may support improved patient selection for OPAT and contribute to optimizing the patient experience of OPAT.

## 4. Conclusions

OPAT is likely to continue to grow in the UK and internationally, driven by a large body of evidence that it is clinically and cost effective, and preferred by patients. The literature review for this update has illustrated the increasing diversity of OPAT services, including an expansion of services based in the community and in acute ambulatory care units.

As with the previous GPRs, further studies have demonstrated the critical importance of a formal OPAT service with a dedicated OPAT team and clear links to organizational governance structures. Complications occur while patients are receiving OPAT and processes must be in place to ensure timely and accurate management. The evidence supports improved outcomes for patients when clinicians with expertise in OPAT have continuing involvement in their care. Furthermore, AMS is now a high priority for healthcare organizations and OPAT has a clear role to play in optimizing this. Unsurprisingly, the literature review included consideration of stewardship in every section of this update. One key consideration is the use of oral therapy in preference to intravenous where appropriate, and OPAT teams may contribute to safe administration of such agents as an extension of their role beyond parenteral therapies.

This update includes some changes from the previous GPRs. Firstly, this update combines both adult and paediatric OPAT in recognition that the principles of safe and effective OPAT are the same in these two groups. When considering the OPAT team, there is a novel concept of the OPAT ‘practitioner’ with a blurring of the professional boundaries between members of the team and a recognition that competence in different aspects of delivering OPAT is not restricted by job title. In patient selection, the literature review highlighted a move away from using rigid selection criteria relating to infection parameters and social factors to a more individualized approach incorporating consideration of comorbidities and recognition that different patient groups may be better suited, or less suited, to specific antibiotics and/or specific delivery models.

With the increasing use of continuous infusion devices there is a need for robust data on the stability of antimicrobial agents, particularly in the ‘real-life’ situation where the device may be maintained near body temperature for prolonged periods. In this update of the GPRs the required standard for stability testing is set deliberately at a high level—that of the BSAC Drug Stability Testing Programme—and we do need to work towards obtaining this level of robust data for a wider range of antimicrobial agents.

In terms of monitoring during OPAT, the requirement for weekly blood tests in patients on prolonged OPAT courses is an evidence-based change from the previous recommendations. Finally, there is the recognition that OPAT may be used in situations where the anticipated outcome is not cure of infection, particularly with increasing use of prosthetic devices in orthopaedics or vascular surgery and use of OPAT for suppression of infection or palliation.

The initial GPRs were intended to serve as a practical resource to help teams to develop or review their OPAT services. This update retains the same practical format and will also serve as a useful summary of the literature relating to OPAT since the publication of the previous recommendations. Additional resources for OPAT services are available at http://www.e-opat.com/; educational resources are available at https://www.futurelearn.com/courses/outpatient-patenteral-antimicrobial-therapy.

## Supplementary Material

dlz026_Supplementary_Data
