# Supplementary material for: Updated good practice recommendations for outpatient parenteral antimicrobial therapy (OPAT) in adults and children in the UK
Source: JAC Antimicrob Resist. 2019 Aug 26;1(2):dlz026. doi: 10.1093/jacamr/dlz026 (PMC8209972; doi:10.1093/jacamr/dlz026)
Supplement: dlz026_Supplementary_Data [file dlz026_supplementary_data.docx]

**Supplementary data**

Table S1. Search criteria used for evidence identification from one of the selected databases (EMBASE).

| **Search Engine** | **#** | **Search term** |
| --- | --- | --- |
| EMBASE | 1 | OPAT.mp. |
|  | 2 | OHPAT.mp. |
|  | 3 | "Outpatient parenteral antibiotic therapy ".mp. |
|  | 4 | "hospital in the home".mp. |
|  | 5 | home infusion therapy/ |
|  | 6 | ((Outpatient or Home) and parenteral and (antibiotic or antimicrobial or antifungal or antiparasitic or antibacterial or antiviral or antiinfective) and (therapy or treatment)).ab. or ((Outpatient or Home) and parenteral and (antibiotic or antimicrobial or antifungal or antiparasitic or antibacterial or antiviral or antiinfective) and (therapy or treatment)).ti. |
|  | 7 | ((outpatient or home) and parenteral and (antibiotic or anti-microbial or anti-fungal or anti-parasitic or anti-bacterial or anti-viral or anti-infective)).ab. or ((outpatient or home) and parenteral and (antibiotic or anti-microbial or anti-fungal or anti-parasitic or anti-bacterial or anti-viral or anti-infective)).ti. |
|  | 8 | "home parenteral therapy program*".ab. or "home parenteral therapy program*".ti. |
|  | 9 | ((outpatient or Home) and (intravenous or IV) and (antibiotic or antimicrobial or antifungal or anti-parasitic or anti-infective or antiviral)).ab. or ((outpatient or Home) and (intravenous or IV) and (antibiotic or antimicrobial or antifungal or anti-parasitic or anti-infective or antiviral)).ti. |
|  | 10 | "Outpatient parenteral antibiotics".ab. or "Outpatient parenteral antibiotics".ti. |
|  | 11 | (outpatient treatment and (intravenous or IV) and (antibiotic or antimicrobial or antifungal or anti-parasitic or anti-infective or antiviral)).ab. or (outpatient treatment and (intravenous or IV) and (antibiotic or antimicrobial or antifungal or anti-parasitic or anti-infective or antiviral)).ti. |
|  | 12 | (community-based and parenteral).ab. or (community-based and parenteral).ti. |
|  | 13 | ("home treatment" and (intravenous or IV) and (antibiotic or antimicrobial or antifungal or anti-parasitic or anti-infective or antiviral)).ab. or ("home treatment" and (intravenous or IV) and (antibiotic or antimicrobial or antifungal or anti-parasitic or anti-infective or antiviral)).ti. |
|  | 14 | ("Home drug infusion" and (antibiotic or antimicrobial or antifungal or anti-parasitic or anti-infective or antiviral)).ab. or ("Home drug infusion" and (antibiotic or antimicrobial or antifungal or anti-parasitic or anti-infective or antiviral)).ti. |
|  | 15 | (Ambulatory and (intravenous or IV or parenteral) and (antibiotic or antimicrobial or antifungal or anti-parasitic or anti-infective or antiviral)).ab. or (Ambulatory and (intravenous or IV or parenteral) and (antibiotic or antimicrobial or antifungal or anti-parasitic or anti-infective or antiviral)).ti. |
|  | 16 | (domiciliary and (intravenous or IV or parenteral) and (antibiotic or antimicrobial or antifungal or anti-parasitic or anti-infective or antiviral)).ab. or (domiciliary and (intravenous or IV or parenteral) and (antibiotic or antimicrobial or antifungal or anti-parasitic or anti-infective or antiviral)).ti. |
|  | 17 | ("Community intravenous antibiotic service" or CIVAS).ab. or ("Community intravenous antibiotic service" or CIVAS).ti. |
|  | 18 | OVIVA.ab. or OVIVA.ti. |
|  | 19 | (IVDU and (antibiotic or antimicrobial or antifungal or anti-parasitic or anti-infective or antiviral)).ab. or (IVDU and (antibiotic or antimicrobial or antifungal or anti-parasitic or anti-infective or antiviral)).ti. |
|  | 20 | 1 or 2 or 3 or 4 or 5 or 6 or 7 or 8 or 9 or 10 or 11 or 12 or 13 or 14 or 15 or 16 or 17 or 18 or 19 |
|  | 21 | clinical competence.mp. or clinical competence/ |
|  | 22 | practice guideline/ or good clinical practice/ |
|  | 23 | health behaviour.mp. or health behavior/ |
|  | 24 | attitude to health.mp. or attitude to health/ |
|  | 25 | health personnel attitude/ |
|  | 26 | 21 or 22 or 23 or 24 or 25 |
|  | 27 | 20 and 26 |
|  | 28 | limit 20 to yr="2010 -Current" |
|  | 29 | limit 28 to human |
|  | 30 | (cancer or childbirth or diabetes).ti. |
|  | 31 | 29 not 30 |
|  | 32 | (Child or children or paediatric or pediatric or infant* or neonate* or babies or toddler* or newborn*).mp. [mp=title, abstract, heading word, drug trade name, original title, device manufacturer, drug manufacturer, device trade name, keyword, floating subheading word] |
|  | 33 | 31 and 32 |
